# The Neighbourhood Method for Measuring Differences in Maternal Mortality, Infant Mortality and Other Rare Demographic Events

**DOI:** 10.1371/journal.pone.0083590

**Published:** 2014-01-02

**Authors:** Nurul Alam, John Townend

**Affiliations:** 1 Centre for Population, Urbanization and Climate Change, ICDDR,B, Dhaka, Bangladesh; 2 Medical Statistics Team, Division of Applied Health Sciences, University of Aberdeen, Aberdeen, United Kingdom; University of California, United States of America

## Abstract

In the absence of reliable systems for registering rare types of vital events large surveys are required to measure changes in their rates. However some events such as maternal deaths are widely known about in the community. This study examined the utility of asking respondents about events in their neighbourhood as an efficient method for measuring relative rates of rare health events such as maternal and infant deaths. A survey was conducted in the health and demographic surveillance system (HDSS) in Matlab, Bangladesh, which includes two areas with different health care regimes. Adult women were asked about any maternal deaths; multiple births; infant deaths, live births and some other events they knew of in a small specified area around their home. Agreement between HDSS records and survey responses was moderate or better (kappa≥0.44) for all the events and greatest for maternal deaths (kappa = 0.77) with 84% being reported. Most events were more likely to be reported if they were recent (p<0.05). Infant mortality rate in one area was 0.56 times that in the other which was well reflected by the ratio of survey results (0.53). Simulations were used to study the ability of the method to detect differences in maternal mortality ratio. These suggested that a sample size around 5000 would give 80% power to detect a 50% decrease from a baseline of 183 which compared well with an estimated sample size around 10 times larger using the direct sisterhood method. The findings suggest that the Neighbourhood Method has potential for monitoring relative differences between areas or changes over time in the rates of rare demographic events, requiring considerably smaller sample sizes than traditional methods. This raises the possibility for interventions to demonstrate real effects on outcomes such as maternal deaths where previously this was only feasible by indirect methods.

## Introduction

There is currently much interest in indicators of health, particularly to measure national progress towards the Millennium Development Goals [1]–[4] yet some of the targets such as a reduction in maternal mortality represent a formidable challenge to measurement [1], [5]. For rare events such as maternal deaths the difficulties arise not only from technical problems of avoiding bias but also from the cost of carrying out sufficiently large surveys to measure the rate per unit time or per birth with reasonable precision. For individual projects the cost of measuring changes in maternal mortality or other rare events, for example following an intervention, is often prohibitively high.

There are a number of existing methods for estimating rates of maternal deaths or the maternal mortality ratio (MMR) in communities with no reliable system for registering vital events [6]. These include reproductive age mortality studies (RAMOS) [7], adding questions to a census [8], [9], direct and indirect sisterhood methods [10], [11], [12], and household surveys [12]. Estimates can also be derived from records kept for a restricted area (e.g. a demographic surveillance system [13]) or from a restricted section of the population (e.g. women giving birth in health facilities [14]).

Whilst all existing methods for measuring MMR have their uses they are all subject to potential biases to varying degrees. However, a bigger limitation to their use is usually the cost and difficulty of applying them in a large enough survey. Say and Pattinson noted that because maternal deaths are a rare event, even very large sample sizes produce estimates of maternal mortality with wide confidence intervals making it difficult to detect differences or changes [15] therefore evaluating an intervention using the above methods is likely to cost hundreds of thousands of US dollars. Costs of adding questions on maternal deaths to another survey (e.g. a census) can be considerably lower but only very large surveys would be suitable so as a means of evaluating an intervention this depends on fortuitous timing. One of the largest maternal health surveys, the Bangladesh Maternal Health and Mortality Survey 2001, obtained estimates of MMR with a 95% confidence interval of around ±15% using the direct sisterhood method and ±20% using a household deaths survey despite using a sample size of approximately 100,000 households [12], [16]. Detecting a *difference* or *change* in MMR is even more problematic because uncertainty in the magnitude of a difference is almost equal to the sum of the uncertainties in the estimates being compared. Thus the cost of making any direct observation of a reduction in maternal deaths is prohibitively expensive for many health programmes and often even for governments. In the Bangladesh survey even an apparent 22% decline in maternal mortality over a 10 year period was not statistically significant with the sample size used. This led Hill et al. [12] to conclude that as a monitoring strategy such surveys cannot be cost effective. The need for more and better data on maternal deaths continues to be stated [17], [18] and there remains a general lack of affordable and convenient methods for measuring changes in MMR or rates of other rare health events such as one might wish to do after trying a health intervention.

The lack of low cost methods for monitoring changes in maternal deaths restricts the number of situations or occasions when direct measurements are made. For this reason associated measures (process indicators such as the rate of Caesarian sections or percentage of births attended by skilled providers), have sometimes been used [19], [20]. However these do not clearly demonstrate a change in maternal mortality and it has been suggested that they should be seen as complementary rather than substitutes where maternal mortality is the target outcome of interest [1].

Some recent developments in the field of measuring maternal mortality have focused on either improving the accuracy of individual measurements using additional information about the area or on using a set of measurements to establish trends [21]–[28] and there has been further attention to refining and improving the efficiency of the sisterhood method [29], [30]. One potential means to reduce the overall cost of measuring MMR is to reduce the cost per interview. This has been investigated by the IMMPACT project which developed a system of Sampling at Service Sites for that purpose [1], [4].

Several recent studies have also demonstrated the potential value of using community knowledge as an efficient method of collecting information. Maskey et al. in Nepal [31] obtained good results eliciting information on maternal and infant deaths using discussion groups comprised of recently delivered mothers and Prata, Gerdts and Gessessew [32] found promising results in a pilot study of a community level reporting system for maternal deaths. Qomariyah et al. [33] used two cross-referenced networks of key informants to identify pregnancy related deaths during the previous 2 years and Barnett et al. [34] used a prospectively established network of key informants to report on births and deaths of women of reproductive age, both studies achieving good estimates of the numbers of deaths. These results correspond with the findings of an earlier study by Boerma and Mati [35] which highlighted the extent to which knowledge of maternal deaths spread within communities in Kenya.

Here we consider a method making use of community knowledge of vital events but without the need to set up a system of meetings and cross-referencing of reports as has been used in other community knowledge based studies. Our method has much in common with traditional survey methods such as direct sisterhood or household surveys but reduces the number of interviews required by increasing the amount of information gathered per interview.

In this study we carried out a survey to test whether adult women's knowledge of nearby vital events in a rural area of Bangladesh would reflect actual events as recorded in a health and demographic surveillance system (HDSS) covering the same area. The demographic surveillance area included two distinct areas where historically the MMR in one area was around 1.6 times that in the other. Although our main interest was in maternal deaths we included other types of events such as child drowning deaths and infant deaths to test the potential of the method. Whilst infant deaths are considerably more common than maternal deaths the cost of measuring the rate through a survey remains appreciable so low cost methods would be useful, particularly to demonstrate the effectiveness of interventions being tested in small scale trials.

The specific objectives of this study were to assess (a) whether adult women know about maternal deaths, infant deaths and other vital events in recent years amongst a wide circle of neighbours, and (b) whether *relative differences* in the MMR and other event rates between the two areas of the HDSS would be accurately reflected in the relative differences calculated from the survey data, even if the reports were not accurate enough to estimate the absolute rates. This should be the case if appreciable over or under-reporting of events was present but consistent for the two areas being compared. Data collection proved to be quite straightforward with village women participating willingly in the interviews and the survey results showed useful associations with actual events recorded by the HDSS.

## Methods

### Ethics statement

Residents of the HDSS area are asked to give written informed consent each year to take part in the data collection activities of the surveillance system which specifically includes collecting information on births and deaths. In addition, for this survey interviews began with the interviewer introducing the objectives and obtaining oral consent from the respondents to participate. The survey was approved by the Ethical Review Committee of the International Centre for Diarrhoeal Disease Research, Bangladesh (ICDDR,B). Considering the nature and context of the study and the fact that the questions do not invade privacy, the committee approved the option of taking oral consent instead of written consent for this survey. Interviewers were instructed not to collect any information unless respondents gave consent therefore completion of a questionnaire was taken as evidence that oral consent was obtained.

### Description of the survey area

The survey was conducted between November 2008 and February 2009 in the Matlab Health and Demographic Surveillance System (HDSS) area in a rural part of Bangladesh about 55 km southeast of Dhaka. There are 142 villages within the study area and within these households are grouped in *baris* sharing a common area with a distinct boundary wall or fence. Baris contain typically around 8 households (inter-quartile range (IQR) 5 to 12 households). The area was chosen for the study because it has a large population under surveillance and a long established, high quality recording system which includes identification of maternal deaths. It is divided into two halves, the *Government* area which receives standard government health care and the *ICDDR,B* area which receives an enhanced level of services [36]. In most years MMR and infant mortality rate (IMR) have been lower in the ICDDR,B area. In 2007, the year before the survey, the MMR (per 100,000 live births) was 156 in the ICDDR,B area and 197 in the Government area whilst IMR (per 1000 live births) was 27.7 in the ICDDR,B area and 39.3 in the Government area [36]. We therefore expected there would be differences in MMR and IMR between the two areas over the study's reference period that we could use to validate the usefulness of our new survey method.

For routine data collection the HDSS is divided into *clusters*, each consisting of around 35 nearby households (grouped into baris) that are visited by one community health research worker (CHRW) in one day during each round of data collection to record vital events and limited child health information. There are 1349 HDSS clusters in total (672 in the ICDDR,B area and 677 in the Government area) with a total population of approximately 224,000. The mean population of a cluster is therefore approximately 166 people, of which around 45 (27%) are reproductive aged women (15–49 years) [36].

### Study Design

A short questionnaire was prepared and the CHRWs were given a half day's training in a group which included discussion of the questions followed by field testing in the same day to finalize the questionnaire. CHRWs were experienced fieldworkers and were told not to let their own knowledge of the area influence the responses. During a normal round of data collection for the HDSS each CHRW visits one cluster each day for 42 days before beginning the cycle again. During one of these rounds CHRWs conducted interviews for this survey in two randomly selected baris of each cluster that they visited. Only baris with four or more households were selected. Smaller baris were excluded as they tend to have little communication with neighbours and the motivation of the project was to develop and test an efficient method of capturing data about events in the community.

In each of the selected baris after routine updating of the HDSS register the CHRW introduced the survey to some of the women and invited any women present in the bari to join in. After those interested had assembled the CHRWs carefully explained the boundaries of the cluster then asked if the women knew of any events of specified types within their cluster during a given reference period. The events asked about in the survey were: any women they knew of who had died whilst they were pregnant, giving birth, or within 6 weeks of a pregnancy ending during the last 3 years; any other women aged 15–49 years who died during the last 3 years; any women who gave birth to twins or triplets during the last 3 years (whether or not the babies survived); any deaths of children aged 10 years or under caused by drowning during the last 3 years; any deaths of infants (aged <1 year) during the last 12 months; and any babies born alive during the last 2 months. Where the respondents answered that they knew of such an event, they were asked to say how many such events they knew of. The reference periods were chosen so that respondents would typically only be trying to recall none, one or two events of each type.

The women present were allowed to discuss the answers between themselves (median 5 women present, IQR 4 – 6) so this approach allowed all interested women present in the bari to take part. A self-appointed spokeswoman, who had to be a married woman aged 18 years or over, coordinated the discussion and was considered as the main respondent for that bari. It was not our intention to test the knowledge of individual women or whether all women knew of events equally well so this was considered to be an efficient way to collect whatever knowledge of local events existed in that community.

All 1349 clusters of the HDSS were included in the survey with two baris selected per cluster and one interview carried out in each of these (total 2698 interviews). This approach achieved a sample of typical village women residents well dispersed throughout the study area. One thousand three hundred and fifty four interviews were carried out in the Government (standard health care) area and 1344 in the ICDDR,B (enhanced health care) area.

### Data Analysis

Data from all 2698 respondents were included as independent reports in the analyses except for the time of events analysis as noted below. The analysis aimed to examine whether reported events corresponded with actual events and also whether *relative* rates of events reported in the ICDDR,B vs. Government areas reflected relative differences in the rates recorded in the HDSS, even if the counts themselves were not accurate.

#### Agreement between reported and HDSS numbers of events

All events asked about in the interviews were also recorded in the routine data collection of the HDSS although in the case of maternal deaths the definition differed. The HDSS records true maternal deaths identified through detailed verbal autopsies whereas, in common with many surveys, we used a time-based definition. Respondents in our survey were asked to report any deaths of women who were pregnant or had been pregnant within 6 weeks of dying. They might therefore have additionally included some accidental or incidental deaths of pregnant or recently pregnant women. For the purposes of the analysis we compared our survey results with the maternal deaths recorded in the HDSS. The implications of this are discussed.

Accuracy of individual reports by respondents was assessed by comparing numbers of events reported in each of the interviews with actual events as recorded by the HDSS in the same cluster. Total numbers of events reported were compared with two times the number of events in the HDSS as there were two respondents within each cluster of the HDSS. We considered events to be correctly reported if there was a corresponding event(s) in the respondent's cluster during the reference period according to HDSS data, under-reported if there was an event(s) in the HDSS that was not included in the response and over-reported if events were mentioned in the response but there was no corresponding event in the HDSS.

Agreement between the numbers of events reported by respondents and the numbers recorded in the HDSS for their cluster and also agreement between the two respondents within the same cluster was assessed using weighted kappa (κ) scores. Agreement can be rated as slight if κ score ranges from 0 to 0.2; fair if κ score ranges from 0.21 to 0.40; moderate if κ score ranges 0.41 to 0.60; substantial if κ score ranges from 0.61 to 0.80; and almost perfect if κ ranges from 0.81 to 1.00.

#### Ability to recall recent or older events

In clusters with exactly one event of a specific type according to the HDSS we investigated whether women's ability to remember the event was affected by how long ago it had occurred. HDSS records were used to calculate the interval between the date of the interview and the date on which an actual event had occurred in that cluster. We assumed for this analysis that if an event was reported it was the same event that was recorded in the HDSS. Clusters with more than one event of the specified type were not included in this analysis because it would not have been possible to tell which event was being reported and hence how long ago it had occurred. The proportion of events correctly mentioned in respondents' answers was calculated for events occurring during different intervals before the survey. Events were grouped into whole years before the survey for maternal deaths, other adult female deaths, child drowning deaths and multiple births, 4 month periods for infant deaths and 20 day periods for live births. Logistic regression was used to test for an association between time since an event in years (calculated to the nearest day) and the odds of it being reported. The analysis was carried out separately for each type of event (maternal deaths, infant deaths, etc.), in each case including all clusters with exactly one event of that type.

#### Estimates of MMR and IMR

Although it was not the main aim of the study the reported numbers of maternal deaths and infant deaths together with multiple births or live births could, in principle, be used to estimate absolute values of MMR and IMR if deaths and births were equally well remembered over the reference periods. To investigate this possibility we calculated MMR using two different assumptions for the number of births in the last 3 years: (i) 18 x reported number of births in the last 2 months, or (ii) reported number of multiple births in the last 3 years/rate of multiple deliveries (irrespective of whether the babies survived). The rate of multiple deliveries per live birth was taken as 0.0102 which was calculated from HDSS records for 2006–2008. Infant mortality rate per 1000 live births (IMR) was calculated similarly except that the survey collected information about infant deaths only in the last 1 year, therefore the numbers of births were one third of those in (i) and (ii) above. Confidence intervals (CIs) for MMR and IMR were calculated using a first-order Taylor series expansion treating the respondents as a random sample from the area, adjusting for similarities between responses within clusters using robust standard errors. MMR and IMR calculated from the survey were compared with the equivalent figures calculated from HDSS data for the same reference periods.

#### Relative rates of events in the ICDDR,B vs Government areas

The main aim of the study was to determine whether the neighbourhood method could be used to detect relative differences or changes in the rates of vital events and in particular in MMR and IMR, comparing two areas with similar sociodemographic characteristics or one area at two different times; a scenario typical of evaluating an intervention. We describe the approach taken in terms of MMR although the same principles could be applied to IMR or some other health events.

The neighbourhood method benefits from the fact that each respondent is providing information about maternal deaths amongst a relatively large number women (rather than just family or household members as in traditional survey methods) so in principle information is gathered very efficiently. However, calculation of absolute values of MMR from our survey data requires assumptions that (i) the net rate of over- or under-reporting is nil or at least the same for both deaths and births, and (ii) that births in the last 2 months or multiple births over the last 3 years are a known fraction of the total live births over the reference period. In practice these assumptions may not be valid and there will usually be no way to check them.

Although it may be difficult to calculate absolute values of MMR reliably with this method we sought to test whether we could use the data collected in the survey to calculate an index which would respond directly to MMR. Using this it should be possible to harness the potential efficiency of the method to compare relative MMRs in two areas, even if the MMRs themselves could not be determined.

We proposed an index which we have called the “neighbourhood index” (*I*).
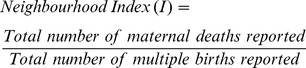



The notion behind this index is that for the area covered by the respondents' neighbourhoods the total number of maternal deaths reported will respond directly to the mean number of maternal deaths per unit area whilst the denominator is expected to respond directly to the mean number of multiple births per unit area. This in turn is expected to be directly related to the mean number of live births per unit area. The index as a whole should therefore be proportional to the number of maternal deaths per unit area divided by the number of live births per unit area, which is the MMR. The exact relationship between the index and MMR will depend on (i) the ratio of live births to multiple births, and (ii) the relative error rates for reporting maternal deaths compared to reporting of multiple births. If it is reasonable to assume that these parameters are the same in two areas then it would then be possible to compare their relative MMRs by comparing the two neighbourhood indices even if the MMRs themselves cannot reliably be determined. Further details of the derivation of the neighbourhood index and the assumptions required to use it for measuring relative differences in MMR are given in Appendix S1.

Neighbourhood indices for maternal mortality and infant mortality were calculated separately for the Government and ICDDR,B areas using our survey data and the above formula. The ratios of neighbourhood indices in these two areas were then compared with the equivalent ratios of MMRs and IMRs calculated from the HDSS data for the same reference periods. If the neighbourhood index for maternal mortality is directly proportional to MMR, as we proposed, the ratio of the indices should be the same as the ratio of the MMRs and similarly for infant mortality.

#### Simulations

In previous years the MMR and IMR had generally been higher in the Government area of the Matlab HDSS and hence we had chosen this as a suitable area for our study to test whether we could detect differences. However, we did not know the actual MMRs over our reference period at the time of conducting the survey because verbal autopsies identifying maternal deaths in the HDSS are carried out some months retrospectively so the total for the last year had not yet been recorded. In the event there was an appreciable difference between the IMRs in the two areas, as in previous years, but the number of maternal deaths over the last 3 years was identical in the two areas so the MMRs were almost the same. Consequently it was not possible to test our hypothesis that the relative difference in MMRs would be reflected by a relative difference in neighbourhood indices. We therefore decided to use simulations based on the data from the HDSS and our survey responses to try to examine the potential ability of the method to detect differences in MMR.

The Matlab HDSS was treated as a reference area and the intention of the simulations was to create a series of comparison areas with varying MMRs but otherwise similar characteristics. In each run neighbourhood type surveys were simulated in both the reference and comparison areas. The ratio of neighbourhood indices derived from these simulated surveys was then calculated to see whether it reflected the relative difference in MMRs between the two areas, as we proposed. The process and rationale for the simulations is described in detail in Appendix S2 and summarized below.

As there was almost no difference in MMR between the Government and ICDDR,B areas over our 3 year reference period the whole of the HDSS was treated as the reference area for all of the simulations. This was divided into 1349 clusters, (as used in the HDSS), 28 of which included a maternal death in the last 3 years. The MMR for the reference area was calculated from the HDSS data for the reference period used in our study and was 183 per 100,000 live births. We refer to this here as the “true” MMR for the reference area as it is based on HDSS records and to distinguish it from results calculated using our survey responses which we refer to as “observed” results.

Each simulation began be creating a new comparison area by randomly selecting 1349 clusters with replacement from the HDSS. This allowed HDSS clusters to be included once, more than once or not at all in the comparison area. In this way each comparison area included a different set of clusters and it was possible to include varying numbers of clusters with a maternal death. Hence comparison areas with differing MMRs could be simulated. In each simulation the “true” MMR for the comparison area was calculated from the HDSS records for those clusters which had been included. If clusters were included more than once the data for these clusters was included the corresponding number of times. A series of 750 simulations were carried out with varying numbers of maternal deaths included in the comparison areas to obtain ratios of MMR (comparison area/reference area) ranging from 0.03 to 2.7.

For the 1349 clusters included in each area we also had 2698 corresponding survey responses from our study (2 per cluster). For any of the HDSS clusters included more than once in a comparison area the corresponding survey responses were included the same number of times. Consequently if a simulated comparison area included more clusters with maternal deaths then correspondingly more of the survey responses were also from clusters with a maternal death. The set of 2698 survey responses in each area was considered to represent the distribution of potential survey responses for women living in that area.

In each simulation we then simulated neighbourhood type surveys in the reference and comparison areas by drawing random samples, with replacement, of 2500 of these potential responses from each area. These were intended to represent plausible results from carrying out neighbourhood type surveys in the reference and comparison areas as these results retain the actual extent of over- and under-reporting found in our real survey and are independent of the HDSS data. The 2500 selected responses were then used to calculate the “observed” neighbourhood index for each area in each simulation run.

In summary, each simulation run therefore generated a “true” MMR for the reference area (always 183), and a “true” MMR for the comparison area – these were calculated from HDSS data for the clusters included in each area. Each run also generated “observed” neighbourhood indices for both the reference and comparison areas calculated from samples of our actual survey responses collected in the corresponding clusters. The ratio of “observed” neighbourhood indices (comparison area/reference area) from each simulation was plotted against the corresponding ratio of “true” MMRs on a scatter graph. The statistical significance of the difference in neighbourhood indices between the reference and comparison areas was calculated for each simulation using standard formulae and these were used to estimate the limit of detection for a difference in MMRs with 80% power (Appendix S2).

The method of drawing samples of survey responses with replacement also allowed us to simulate larger samples with the same realistic distributions to examine the effect of sample size on detectable ratios of MMR. Further series of simulations were carried out using samples with 5000, 10,000, 20,000 and 40,000 respondents per area. For comparison the relative differences in MMR detectable using a direct sisterhood type questionnaire in the same area were also estimated. The number of sisters of reproductive age per respondent was assumed to be 2 (the approximate number found in the Bangladesh Maternal Death survey 2001 [16]). General fertility rate (GFR) was assumed to be 83 per 1000 women aged 15–49 per year [36] and we assumed that perfect information on years of exposure and sisters' deaths was obtained from independent, randomly selected respondents for a three year reference period. Detectable ratios of MMR with 80% power for both the neighbourhood and direct sisterhood type surveys were plotted against sample size to compare the sensitivity of the two techniques.

Analyses were carried out using Stata v12 (Stata Corp., TX).

## Results

### Agreement between reported and HDSS numbers of events

The proportion of reported events that corresponded with actual events in the HDSS varied from 48% for infant deaths to 74% for live births in the last 2 months (maternal deaths  = 71%) (Table 1). Between 16% (maternal deaths) and 55% (non-maternal 15–49 year old female deaths) of HDSS events went unreported. Over-reporting (reporting of events with no corresponding event in the HDSS) ranged from 26% of reported events for live births to 52% for infant deaths. Overall 1942 (60%) out of a possible 3238 events of various types were correctly reported and 68% of events that were reported corresponded with events in the HDSS. The weighted kappa score for agreement between reports and HDSS totals was at least 0.44 (moderate agreement) for all of the types of vital events and was substantial for maternal deaths and drowning deaths. For all of the types of events the correspondence between reports and HDSS records was significantly better than chance (p<0.001). The 1296 events that were missed were largely balanced by 896 reported events that did not correspond with events in the HDSS records (over-reports), making the overall number of events reported only 12% below that in the HDSS. For individual types of events the overall totals reported ranged from 68% of the HDSS total for non-maternal 15–49 year old female deaths to 118% of the HDSS total for maternal deaths.

**Table 1:** Accuracy of reporting of events in the respondents' own clusters and agreement with HDSS records and other respondents in the same cluster.

	HDSS total	Un-reported		Total reported		Correct reports		Over-reports		Weighted κ score	
	n	n	%	n	%	n	%	n	%	Between HDSS and survey	Between respondents
Maternal deaths	56	9	(16)	66	(118)	47	(71)	19	(29)	0.77	0.75
Other adult female deaths	414	227	(55)	282	(68)	187	(66)	95	(34)	0.48	0.65
Drowning deaths	250	66	(26)	273	(109)	184	(67)	89	(33)	0.68	0.69
Infant deaths	294	150	(51)	297	(101)	144	(48)	153	(52)	0.44	0.47
Multiple births	314	128	(41)	306	(97)	186	(61)	120	(39)	0.55	0.66
Live births	1910	716	(37)	1614	(85)	1194	(74)	420	(26)	0.50	0.54
All events	3238	1296	(40)	2838	(88)	1942	(68)	896	(32)		

HDSS total  =  number of events that should have been reported (number of events in the HDSS x 2 respondents per cluster).

Un-reported  =  events missed by respondents (% of HDSS total).

Total reported  =  number of events reported (% of HDSS total) (includes correct reports and over-reports).

Correct reports  =  events reported that corresponded with actual events (% of total reported).

Over-reports  =  events reported over and above the actual number of events in the respondents' clusters (% of total reported).

All weighted κ scores were highly significant (p<0.001).

Agreement between the two responses per cluster was approximately the same or better than agreement between the survey responses and the HDSS records (Table 1).

### Ability to recall recent or older events

The time since an event occurred was negatively associated with the odds of it being reported for other 15–49 year old female deaths, child drowning deaths, infant deaths and multiple births (p<0.05) (Table 2). The odds of an infant death being reported fell most rapidly with time (OR 0.42 per year) although this had less effect on our survey results as the reference period was only one year compared with other 15–49 year old female deaths which fell from 64% being reported if the event occurred in the last year to 28% if it occurred between 2 and 3 years previously (OR 0.49 per year). Although the highest rate of reporting of maternal deaths was for events that occurred in the year preceding the survey there was no clear trend with time with over 80% being reported even if they had occurred 2 to 3 years previously. Reporting of live births according to time before the survey did not follow any obvious pattern but the highest rate of reporting (70%) was for births 20 to 39 days before the survey.

**Table 2:** Respondents reporting an event in clusters where exactly one event had taken place during the reference period.

Event	OR	(95% CI)	p-value	Time before survey	n	/ N	(%)
Maternal death	0.51	(0.22, 1.20)	0.124	<1 year	26	/ 28	(93%)
				1 - <2 years	11	/ 16	(69%)
				2 - <3 years	10	/ 12	(83%)
				total	47	/ 56	(84%)
Other 15–49 y old female death	0.49	(0.38, 0.64)	<0.001	<1 year	73	/ 114	(64%)
				1 - <2 years	58	/ 114	(51%)
				2 - <3 years	34	/ 122	(28%)
				total	165	/ 350	(47%)
Child (10 y or under) drowning death	0.60	(0.41, 0.89)	0.010	<1 year	44	/ 54	(81%)
				1 - <2 years	58	/ 74	(78%)
				2 - <3 years	49	/ 78	(63%)
				total	151	/ 206	(73%)
Infant (under 1 y) death	0.42	(0.18, 0.96)	0.040	<4 months	52	/ 98	(53%)
				4 - <8 months	37	/ 80	(46%)
				8 - <12 months	33	/ 80	(41%)
				total	122	/ 258	(47%)
Multiple birth	0.65	(0.49, 0.87)	0.004	<1 year	57	/ 82	(70%)
				1 - <2 years	83	/ 134	(62%)
				2 - <3 years	30	/ 60	(50%)
				total	170	/ 276	(62%)
Live birth	4.93	(0.29, 83.20)	0.268	<20 days	176	/ 307	(57%)
				20 - <40 days	194	/ 279	(70%)
				40 - <60 days	174	/ 278	(63%)
				total	544	/ 864	(63%)

OR =  odds ratio (per year before the survey) for an event being reported (OR<1 means the event was less likely to be reported the greater the time it occurred before the survey).

n =  number of respondents correctly reporting the event.

N =  number of clusters with exactly one event of this type during the reference interval (x 2 respondents).

% =  % of potential reports included in responses.

### Estimates of MMR and IMR and comparisons of areas

From the survey data estimates of MMR and IMR calculated using reported multiple births were 220 (95% CI 140 – 300) and 29.7 (95% CI 24.0 – 35.4) and when calculated using live births in the last 2 months the estimates were 227 (95% CI 151 – 304) and 30.7 (95% CI 25.7 – 35.6) respectively. These can be compared with the equivalent figures calculated from the HDSS data for the reference periods of our survey: MMR = 183 per 100,000 and IMR = 29.1 per 1000 live births. In both cases the estimate using multiple births was closer to the HDSS figure. Using a sample size of 2698 MMR was estimated with a 95% CI of approximately ±36% whilst IMR was estimated with a 95% CI of approximately ±19%. Neither was significantly different from the figure calculated using HDSS data.

The ratio of MMRs in the two areas according to the HDSS for our reference period was close to one (0.96; ICDDR,B/Government). In comparison the ratio of neighbourhood indices calculated from our survey data for maternal deaths was 0.67. For IMR the ratio of the two areas in the HDSS was 0.56 which compared well with the ratio of neighbourhood indices we calculated from our survey data for infant deaths of 0.53.

### Simulations

**Figure 1:**
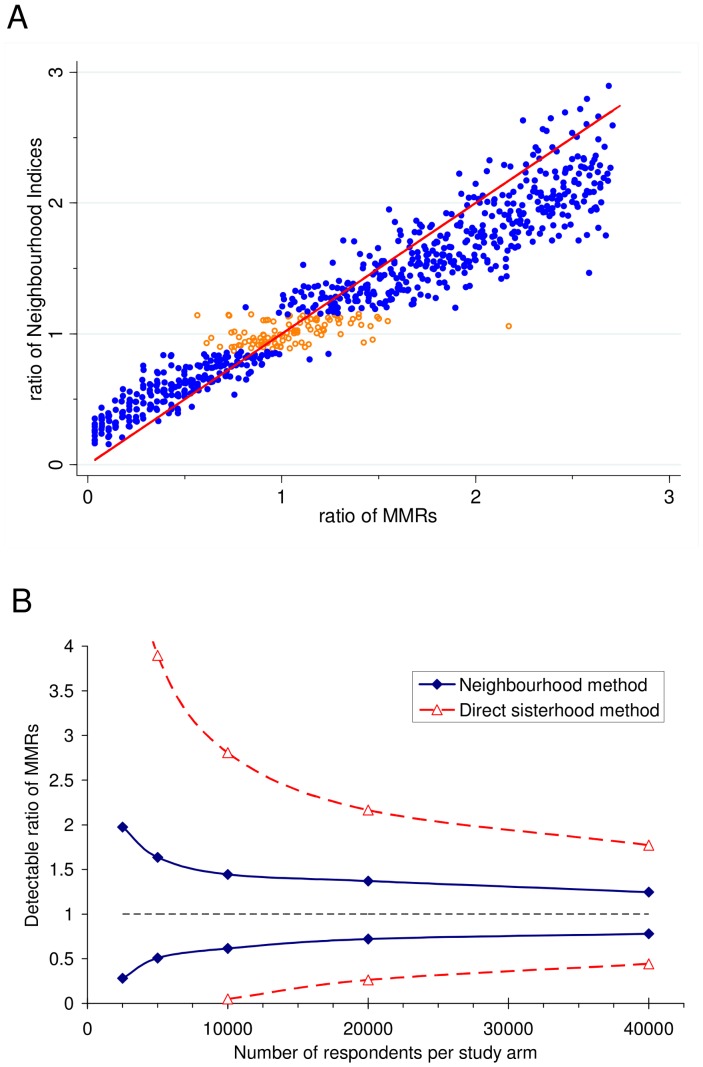
Results of simulations. (A) Ratios of observed neighbourhood indices for a comparison area and the reference area (*I*_comp_/*I*_ref_) compared with ratios of true MMRs (MMR_comp_/MMR_ref_) for a sample size of 20,000 per area. Each point represents the results of one simulation. In each case the MMR in the reference area was 183 per 100,000 live births. Ratios of neighbourhood indices which were significantly different (p<0.05) are shown in blue (filled circles); non-significant results are shown in orange (open circles). 1:1 line is also shown. (B) Detectable differences – the lines show ratios of MMR_comp_/MMR_ref_ which could be detected with 80% power at the 5% significance level for a given sample size when MMR_ref_ = 183. The equivalent curves using a direct sisterhood survey with reference period 3 years assuming 2 sisters per respondent, GFR = 83 and perfect information from survey responses are also shown.

The “observed” ratios of neighbourhood indices showed a clear linear association with the “true” ratios of MMRs in the simulated surveys (Figure 1A). For all sample sizes the slope of the graph was slightly less than unity suggesting that the neighbourhood method would be conservative in measuring the true relative change or difference in MMR. This association between the ratio of neighbourhood indices and the ratio of MMRs became stronger as the sample size increased so that the detection limits became narrower (Figure 1B). These compared well with the expected limits for detecting differences using the direct sisterhood method for the same area and sample sizes. The simulations suggested that a sample size of around 5000 would be sufficient to detect a 50% fall in MMR from a baseline value of 183 per 100,000 with 80% power and we estimated that a sample size around 52,000 would have been required to detect the same change in an area with similar demographic characteristics using the direct sisterhood method. For sample sizes of 10,000 and 20,000 the simulations suggested that it would be possible to detect a 38% decrease or a 28% decrease in MMR respectively using the neighbourhood method (with 80% power) in this setting.

## Discussion

Our study was focused on developing an efficient survey method for measuring relative differences in MMR or IMR or other rare health events using respondents' knowledge of events in their neighbourhoods. All survey methods for measuring maternal and other types of deaths suffer to some extent from omission of deaths and dating errors [37] although it is likely that more events will be missed when asking about deaths or births outside of a respondent's immediate family or household. In this study we found that many vital events were well known about within the community although there were also numerous over-reports. One possibility is that some of the over-reports were real events that occurred outside the time frame or area that the respondents were asked to include. This might account for why weighted kappa scores for agreement with other respondents in the same cluster were greater than agreement with the HDSS data for non-maternal 15-49 year old female deaths and multiple births.

Under-reporting was largely counterbalanced by over-reporting in this survey and consequently absolute estimates of MMR and IMR were not significantly different from the HDSS figures. However, we have no reason to assume that these two influences would be similar in other applications and therefore it would be difficult to use this method for *absolute* measurements of rates or ratios of maternal or other deaths without some evidence of the extent of over- and under-reporting that had occurred.

Maternal deaths seemed to be particularly well known about; only 9 out of a possible 56 reports (16%) were missed by respondents. There were also 19 over-reports of maternal deaths. It is possible that some of these were other adult female deaths that were thought to be maternal and we can also not rule out the possibility that some were genuine maternal deaths that were not identified by the HDSS (e.g. abortion related deaths that women did not want to discuss with HDSS staff). Our survey used a time related definition for the questions about maternal death – death of a woman while pregnant or within 42 days of termination of pregnancy, irrespective of the cause. These are more correctly called *pregnancy related deaths* [1], [15]. The HDSS system, conversely, records true maternal deaths (i.e. excluding deaths from accidental or incidental causes within the time frame). Hence some of the apparent over-reporting may also be due to differences in definition. It is estimated that about 15% of pregnancy related deaths are not true maternal deaths [12], [38]. We also noted that 12 of the 19 over-reports were recorded by just two of the 38 CHRWs. Both of these had worked in the Government area and the over-reporting in their clusters resulted in an apparent difference in neighbourhood indices between the two areas although there was little difference in the real MMRs. These over-reports might reflect the way that these two CHRWs were asking the questions (e.g. inadvertently eliciting information about events from nearby clusters) and highlights the need for careful training and review for the method to be used successfully.

It is likely that the accuracy of reporting would be related to the number of events respondents are being asked to recall which in turn is related to the length of reference period. In our study the reference periods were chosen so that we expected women would be trying to recall none, one or two events of each type however live births were so frequent that 4% of respondents were being asked to report more than 2 events during the last 2 months. Reducing the time frame to <1 month would not necessarily have helped as we found that the greatest proportion of births were reported if they occurred 20–39 days before the survey. This may reflect the time it takes for news to spread around a neighbourhood. In general though, more recent events were better reported so there is scope to improve accuracy by using shorter reporting periods. However, as with methods like the direct sisterhood and household surveys reducing the length of reference period would mean increasing the sample size required to cover the same number of women years at risk and hence achieve the same precision in the estimates.

The size of neighbourhoods and clarity of the boundaries may also have an effect. The neighbourhoods used in this study were chosen to coincide with units used in the routine HDSS data collection and consisted of around 35 households (circa. 166 people). These were used so that we could verify the reports against HDSS records. Different definitions of neighbourhoods may be more appropriate in other settings (e.g. village) however, in larger villages respondents may not know about the whole of the village and the boundaries may be less clear. The use of a “neighbourhood index” to compare areas was conceived to try to overcome this. This index relates the number of maternal (or other) deaths reported in an area to the number of multiple births. If knowledge of deaths diminishes at a similar rate to multiple births with increasing distance, in principle it is not necessary to use neighbourhoods with well-defined boundaries. Neighbourhoods can have a natural boundary which is the range of a respondent's knowledge of events. Totaled over all respondents the ratio of deaths to multiple births reported should be the same whether individual respondents have large neighbourhoods or small, provided the neighbourhoods are contained within the area of interest for the study. Although it is probably only feasible to ask about pregnancy related deaths rather than true maternal deaths this would also not affect the ratio of neighbourhood indices if the proportion of accidental and incidental deaths can be assumed to be the same in both areas. The ratio of two neighbourhood indices (neighbourhood index = total deaths reported/total multiple births reported) from sociodemographically similar areas should therefore be an estimate of the ratio of their MMRs or IMRs. Thus a ratio significantly different from one would imply different MMRs or IMRs.

Measuring both the numerator (maternal or infant deaths) and denominator (multiple births) in the same survey adds a degree of robustness to this method although certain assumptions are needed. Asking respondents to remember any live births in the last 3 years would be too difficult so it must be assumed that the twinning rate is the same (or known) in the areas or times being compared. This is probably reasonable, at least for the few years over which one might want to measure the impact of an intervention. Alam et al. [39] found the twinning rate (proportion of deliveries that were multiple births) in Matlab had stayed approximately constant at around 1% over the 27 year period 1975 – 2002. It must also be assumed that the percentage of over- and under-reporting is the same in both areas (although not that over- and under-reporting are themselves equal or that they are the same for deaths and multiple births). In our survey although 51% of infant deaths went unreported and 52% of those that were reported were over-reports, these effects were very similar in the two areas so that the calculated ratio of neighbourhood indices (0.53) was very close to the ratio of IMRs that we aimed to represent (0.56). It was somewhat surprising that over-reporting of maternal deaths was more common in one area than the other since maternal deaths on the whole were reported quite accurately. However, this may have been an issue of training of survey staff as noted above. Using the same survey staff in both areas might help to overcome any such possible biases.

Our study design, using two respondents per cluster, was chosen so that an acceptable number of interviews could be carried out within the HDSS area and had the advantage that we could compare responses within clusters. In practice it would be unnecessary and inefficient to interview respondents with overlapping neighbourhoods therefore sampling methods that achieved a well dispersed set of respondents would be desirable. This might be achieved by piggy-backing such a survey on to another survey covering the area of interest or by using the sampling at service sites approach to interview women coming from a wide area to a market or health centre [1], [4].

The fact that reports of maternal deaths, infant deaths, multiple births, and other events corresponded with HDSS records to an extent better than chance demonstrates that the method could discriminate between areas with high and low MMR or other rates if large enough sample sizes were used. However, our purpose in testing the method was to develop a way of detecting changes or differences in MMR and other vital rates with smaller samples than existing survey methods. To estimate the detectable difference that might be achieved using different sample sizes in a similar setting we used simulations, treating the clusters of the HDSS as a typical sample of “neighbourhoods” from such an area and the responses from the survey as a typical set of reports that would be obtained using this questionnaire. Simulations cannot incorporate all of the influences that might occur in real life but the results suggested that a survey of around 20,000 people would have 80% power to detect a fall of 28% or an increase of 37% in MMR from a base level of 183 which compared favourably with expected detection limits of a 74% decrease or a 117% increase using the same sample size with the direct sisterhood method. Even a sample size of 5000 was sufficient to detect a 50% decrease in MMR using the neighbourhood method (compared with around ten times larger using the direct sisterhood method). In areas with a higher baseline MMR it can be expected that smaller sample sizes would be needed to detect the same relative increase or decrease.

Despite imperfect information this gain in efficiency results from the considerably greater number of women years at risk covered by each interview when using the neighbourhood method. Increasing the sample size to 40,000 for the neighbourhood survey did not reduce the detection limits much further. There is probably a practical limit to the difference that could be detected with this method which would depend on the extent of random errors in the reports themselves. In the simulations the probability of a death or birth being reported in a cluster where none had occurred was constant and equal to that found in our actual survey. In a real survey the rate of over-reporting might be proportional to the rate that events were occurring in the community in general in which case the number of over-reports would be reduced in areas with lower MMR. This would have the effect of making the technique more sensitive for detecting changes or differences in MMR than we found in these simulations.

The need for good quality data to drive decision making has been stated many times [1], [40], [41] although Horton [42] noted that lack of reliable data continues to hamper country efforts to address problems of maternal mortality with a third of the necessary data either lacking or unusable. He therefore urged that it should be a funding priority to support country-led continuous monitoring of health programmes. Graham et al. highlighted the increased difficulty when measuring *changes* in rates of maternal deaths [1]. In a review of 109 studies of interventions aimed at reducing maternal deaths in low-income countries, maternal death was measured in only 48 studies and almost all sample sizes were too small to detect significant differences [20].

The method we describe here does not aim to compete with established methods for measuring maternal mortality or other vital events but rather to supplement the range of methods available and may be particularly useful for demonstrating a change in MMR or IMR. For projects with a limited budget it may provide a viable option where none previously existed and could, for example be used alongside process indicators to provide a demonstration that an intervention has had a real impact on maternal deaths. In principle the neighbourhood method requires only two questions to measure differences in maternal mortality or infant mortality – “how many deaths can you recall?” and “how many multiple births can you recall?” – for a stated area and reference period. The questions could therefore easily be added to another survey as even modest sample sizes could give useful estimates of change.

The main assumption in the neighbourhood method for detecting differences in MMR, IMR or other health outcomes is that the error rates amongst reports of the events of interest would be the same in the two areas or two times being compared. This may not hold true if, for example, a publicity campaign was launched concerning maternal deaths. However, it would usually be possible to predict the direction of any such bias (e.g. it seems likely in this case that the estimate of any reduction in MMR would be conservative because there would be proportionately more reporting of maternal deaths in the intervention area). So far we have only been able to test the method thoroughly in one setting and this may have been atypical in some respects. Respondents were part of the HDSS and therefore accustomed to answering health related questions. Furthermore the survey staff were familiar with the respondents and with many of the actual events. Although they were told not to let their own knowledge influence the responses we cannot rule out this possibility. Finally social structures determining the extent of knowledge of events in the local community might be unique to this area. Further studies are therefore needed to test whether this would be an effective method in other settings.

## Supporting Information

Appendix S1 **Derivation of the Neighbourhood Index.**
(DOC)

Appendix S2 **Simulations to compare areas with differing MMR.**
(DOC)
