# Supplementary material for: The Neighbourhood Method for Measuring Differences in Maternal Mortality, Infant Mortality and Other Rare Demographic Events
Source: PLoS One. 2014 Jan 2;9(1):e83590. doi: 10.1371/journal.pone.0083590 (PMC3879230; doi:10.1371/journal.pone.0083590)
Supplement: Appendix S1 — Derivation of the Neighbourhood Index. (DOC) [file pone.0083590.s001.doc]

**Appendix S1**

Derivation of the Neighbourhood Index

Existing methods for measuring MMR can be used to measure a difference in MMR because two measurements can be made in different areas and compared. This requires both measurements to be made with sufficient precision and this usually requires large sample sizes and hence large amounts of money and/or resources. Hence comparisons of the MMR in two areas (e.g. to evaluate an intervention) are often unaffordable using existing methods even when they are technically feasible.

However, detecting a difference in MMRs does not in itself require either MMR to be known. We could, for example, demonstrate that the MMR in one area is half that in the other without knowing either MMR. Whilst this imposes some restrictions on where such a method will be useful it also offers some opportunities to make the necessary measurements *via* a more efficient process. With this in mind an index was developed which could be calculated from respondents’ knowledge of events in their neighbourhood and is directly proportional to the MMR in an area. Its exact relationship to MMR depends on certain sociodemographic characteristics of the area which will generally be unknown. However, provided these can be considered the same in the two areas the relative MMRs could be compared by comparing two indices without actually quantifying these characteristics. The description below concerns the comparison of MMRs in two sociodemographically similar areas although the technique might equally be applied to comparing the MMRs in one area at two times (e.g. before and after an intervention) and the method might also be applied to comparing IMRs or some other health indicators.

MMR is defined as:

$$MMR=\frac{D}{B}$$

where *D* is the number of maternal deaths in the study area and *B* is the number of live births in the study area over the same period. (In practice MMR is usually quoted per 100,000 live births to give more convenient numbers).

Within the study area we might also state this as:

$$MMR=\frac{D'}{B'}$$

where *D’* is the number of maternal deaths per unit area per unit time and *B’* is the number of live births per unit area per unit time over the same reference period.

Our survey method involves asking respondents about events they know of in their neighbourhood rather than just in their family or household. Hence each respondent can potentially report on a greater number of women and data on maternal deaths can be captured more efficiency than by traditional survey methods. A trade off in this will be less accuracy in individuals’ reports which we consider below.

Let each survey respondent, *i*, have a neighbourhood with area *a_i_* which may be defined to be approximately the same size for all respondents (e.g. their HDSS cluster in the present study) or may vary (e.g. the respondent’s home village, which will depend on the size of their village).

Because maternal deaths are fairly rare it will typically be necessary to ask respondents about any maternal deaths they know of in their neighbourhood over the last 1 to 5 years before the survey. Over this period the number of births in respondents’ neighbourhoods will probably be too large to remember. A more unusual but related event is a multiple birth. Let *k* be the ratio of multiple births to live births. The density of multiple births per unit area per unit time in the study area, *M’*, is therefore:

$$M'=k B'$$

Respondents in the survey were asked to report any maternal deaths and multiple births they could remember in their neighbourhood over a period, *t* years, before the survey (the last three years in our survey). The total numbers of maternal deaths, *R*, and multiple births, *S*, they *should* report are therefore:

$$R=\sum\left(a_{i}\right) D' t$$

$$S=\sum\left(a_{i}\right) M' t=\sum\left(a_{i}\right) k B' t$$

In reality we would expect some proportion of under-reports (omissions) and also some proportion of over-reports due to mistakes and inclusion of events from outside the specified time period or neighbourhood. Let the net reporting rates for maternal deaths and multiple births amongst the respondents be *υ* and *τ* respectively. Note that these will be greater than one if over-reporting exceeds under-reporting and less than one if over-reporting is less than under-reporting. Note also that *υ* and *τ* are not necessary equal because maternal deaths may be better or less well remembered than multiple births.

Our index, which we have called the neighbourhood index (*I*), was defined as the ratio of total number of maternal deaths actually reported divided by the total number of multiple births actually reported:

$$I=\frac{\sum\left(a_{i}\right) D' t \upsilon}{\sum\left(a_{i}\right) k B' t \tau}$$

$$I=\left(\frac{\upsilon}{k \tau}\right)\times\left(\frac{D'}{B'}\right)$$

$$I=\left(\frac{\upsilon}{k \tau}\right)\times MMR$$

Subscripting with 1 for one area and 2 for a second area to be compared with it, the ratio of the neighbourhood indices for two areas is:

$$\frac{I_{1}}{I_{2}}=\frac{\left(\frac{\upsilon_{1}}{k_{1} \tau_{1}}\right)\times MMR_{1}}{\left(\frac{\upsilon_{2}}{k_{2} \tau_{2}}\right)\times MMR_{2}}$$

If $\left(\frac{\upsilon_{1}}{k_{1} \tau_{1}}\right)=\left(\frac{\upsilon_{2}}{k_{2} \tau_{2}}\right)$then:

$$\frac{I_{1}}{I_{2}}=\frac{MMR_{1}}{MMR_{2}}$$

The ratio of the two neighbourhood indices is therefore equal to the ratio of the two MMRs provided that *υ*/(*k* *τ*) can be assumed to be constant.

The method therefore requires two assumptions:

1. The twinning rate is the same in the two areas being compared, hence *k*_1_ = *k*_2_, or very nearly so. This assumption seems reasonable when comparing two sociodemographically similar areas or one area before and after an intervention over the relatively small number of years one would normally wish to do this. Alternatively if the twinning rates in the two areas are different but known the ratio of Neighbourhood Indices could be adjusted accordingly.
2. Relative ability to report maternal deaths and multiple births is the same in both areas (i.e. *υ*_1_ / *τ*_1_ = *υ*_2_/ *τ*_2_). As the method is intended for comparing sociodemographically similar groups of respondents it seems reasonable to suppose that net reporting rates will be the same in both areas, or very nearly so (i.e. *υ*_1_ = *υ*_2_ and *τ*_1_ = *τ*_2_) so this assumption would be met. An exception to this would be if there are more responses from larger villages in one area then one might expect more omissions of maternal deaths and hence a lower net reporting rate. However one could expect a similar proportionate increase in omissions of multiple births due to the larger neighbourhoods. Hence the assumption *υ*_1_ / *τ*_1_ = *υ*_2_/ *τ*_2_ would still be met.

These are important limitations of the method and must be borne in mind whenever it is proposed to use it. However, since evaluations of an intervention or simply the study of changes over time will usually involve comparing sociodemographically similar areas we anticipate that the assumptions would be reasonable for many situations where the method is likely to be used.
