# Supplementary material for: The Neighbourhood Method for Measuring Differences in Maternal Mortality, Infant Mortality and Other Rare Demographic Events
Source: PLoS One. 2014 Jan 2;9(1):e83590. doi: 10.1371/journal.pone.0083590 (PMC3879230; doi:10.1371/journal.pone.0083590)
Supplement: Appendix S2 — Simulations to compare areas with differing MMR. (DOC) [file pone.0083590.s002.doc]

**Appendix S2**

Simulations to compare areas with differing MMR

The Matlab HDSS area is divided into fixed non-overlapping “clusters” each of which contains around 35 households that would be visited by one CHRW in one of the normal rounds of data collection. As there was almost no difference in the MMR for the Government and ICDDR,B areas over our reference period, data from the whole Matlab HDSS were pooled (1349 clusters) and this was considered a reference area for our simulations. HDSS records include the number of maternal deaths and live births in each cluster over the last three years and from these we calculated that the MMR in our reference area for our reference period was 183 per 100,000 live births, which we refer to here as the “true” MMR in the reference area.

Each simulation run involved two stages:

1. Simulation of a comparison area
2. Simulation of a neighbourhood type survey in the reference and the comparison areas
3. *Simulation of a comparison area*

In the reference area 28 of the 1349 clusters included a maternal death whilst no clusters had more than one maternal death. A new comparison area was created for each simulation consisting of a bootstrap sample of *n* of the clusters where a maternal death had occurred and 1349-*n* of the clusters where no maternal deaths had occurred. Bootstrap samples are drawn at random from an original sample with replacement, meaning that any cluster could be drawn once, more than once or not at all. Hence it was possible to vary *n* in different simulations to give comparison areas with a range of numbers of maternal deaths. On average, a set of bootstrap samples retain the distribution of data in the original sample so in effect we simulated comparison areas with 1349-*n* clusters typical of those that did not contain a maternal death and *n* clusters typical of those that did. In this way we were able to simulate comparison areas with a range MMRs which were otherwise very similar to the reference area.

Each cluster in a comparison area corresponded to a cluster in the HDSS although not all of those in the HDSS were included and some were included more than once. For each of the included clusters we had the HDSS data on the number of maternal deaths and live births in the last 3 years. If HDSS clusters were included more than once in the comparison area so was the count of maternal deaths and live births in that cluster. Hence it was possible to calculate the MMR for the simulated comparison area over the reference period. As this was calculated only from HDSS data we refer to this here as the “true” MMR for the comparison area to distinguish it from results based on our survey responses (see below).

1. *Simulation of a neighbourhood type survey in the reference and the comparison areas*

Both the reference and comparison areas consisted of 1349 of the HDSS clusters and for these there were 2698 corresponding survey responses (two from each of the clusters included). For the comparison area, if HDSS clusters were included more than once the corresponding survey responses were also included more than once. Therefore for simulated comparison areas with more maternal deaths proportionately more of the responses included were from women in clusters where there had been a maternal death.

In our study we collected two independent responses from each cluster to simplify the logistics of data collection and to allow comparison of responses within a cluster. However, in most real world applications we would envisage that responses will be collected from random samples of women in the reference and comparison areas. We therefore simulated surveys by drawing samples of 2500 from amongst the 2698 responses in each area using bootstrap sampling. This is equivalent to treating the 2698 responses from each area as representing the distribution of potential responses in a large population and selecting a sample of 2500 at random from that area.

On average, a set of bootstrap samples retain the distribution of data in the original sample whilst allowing for the variability that would be obtained by repeatedly drawing random samples of this size. As the results were drawn from real survey responses, on average the real extent of over- or under-reporting found in our study was also preserved in the simulated survey results. By running a series of such simulations we therefore obtained sets of plausible results that might have been obtained by carrying out neighbourhood type surveys in the reference and comparison areas.

For the 2500 sampled responses in each area we calculated the neighbourhood index (total number of maternal deaths reported / total number of multiple births reported). We refer to these as the “observed” neighbourhood indices as they are calculated from samples of our actual survey results without reference to the HDSS data.

A series of 750 simulations were carried out, varying the number of clusters with a maternal death in the comparison areas to obtain simulated ratios of MMR (comparison area / reference area) ranging from 0.03 to 2.7. In each simulation the ratio of the “observed” neighbourhood indices, *I_Comp_* /*I_Ref_* was calculated and these were compared with the corresponding ratios of “true” MMRs using a scatter graph.

For each simulation we tested whether there was a significant difference between the “observed” neighbourhood indices for the comparison vs. reference areas using standard statistical formulae and methods. Standard errors for each of the “observed” neighbourhood indices were calculated using first-order Taylor series expansion and the pooled standard error for the difference between “observed” neighbourhood indices was then calculated as the square root of the sum of the two squared standard errors. This was used to test the significance of the null hypothesis *I_Ref_* = *I_Comp_* using a z-test. A p-value < 0.05 was taken to indicate a statistically significant difference. Using data from the 750 simulations a logistic regression model predicting the odds of a significant difference between “observed” neighbourhood indices was fitted as a function of the ratio of “true” MMRs. This model was then used to estimate the smallest ratio >1 and the largest ratio <1 that would produce a significant difference in “observed” neighbourhood indices 80% of the time (i.e. with 80% power).
